# Social connection and substance use behavior among college students

**DOI:** 10.1192/j.eurpsy.2026.10233

**Published:** 2026-07-31

**Authors:** C. Verledens, F. Gericke, L. Jansen, R. Bruffaerts

**Affiliations:** 1 Center for Public Health Psychiatry, KU Leuven, Leuven, Belgium; 2 Institute for Life Course Health Research, Stellenbosch University, Stellenbosch, South Africa; 3 UPC KU Leuven, Leuven, Belgium

## Abstract

**Introduction:**

The transition to higher education often involves moving away from home and navigating a new social environment. For college students, maintaining pre-existing interpersonal relationships while establishing new social networks presents notable challenges. Successfully fostering social connection has been linked to improved health outcomes, including reduced vulnerability to risky substance use behavior. Nevertheless, substantial variability in the conceptualization of social connection hampers clear insights into its association with substance use behavior and limits the development of targeted prevention and intervention strategies.

**Objectives:**

This study aims to (1) establish a clear conceptualization of social connection among college students, and to. (2) examine the association between social connection and substance use behavior.

**Methods:**

We analyzed data from a representative sample of Flemish college students (n = 21,313; R-indicators of 94%) who participated in the Flemish College Surveys, an online survey conducted between 2022 and 2023. The survey was based on the Composite International Diagnostic Interview Screening Scales (CIDI-SC) which included items on social network characteristics, positive and negative aspects of interpersonal relationships, and substance use behavior. Exploratory (EFA) and confirmatory factor analyses (CFA) were performed to identify the underlying structure of social connection. Subsequently, we examined the unique associations between each factor and substance use behavior.

**Results:**

Through EFA we identified a five factor structure. The factors could be labeled as (1) online network, (2) offline network, (3) social support, (4) social strain, and (5) loneliness. Our analyses indicate that these factors are related to substance use, with substance use being related to denser online and offline networks, lower social support, higher social strain, and higher loneliness.

**Conclusions:**

Given the observed associations between social connection and substance use, targeting specific aspects of social connection may enhance the effectiveness of prevention and intervention programs aimed at reducing substance use among college students.

**Disclosure of Interest:**

None Declared

